# A comparison of qSOFA, SIRS and NEWS in predicting the accuracy of mortality in patients with suspected sepsis: A meta-analysis

**DOI:** 10.1371/journal.pone.0266755

**Published:** 2022-04-15

**Authors:** Can Wang, Rufu Xu, Yuerong Zeng, Yu Zhao, Xuelian Hu

**Affiliations:** 1 Department of Pharmacy, The Second Affiliated Hospital of Army Medical University, Chongqing, China; 2 Department of Pharmacy, University Town Hospital Affiliated of Chongqing Medical University, Chongqing, China; Babol University of Medical Science, ISLAMIC REPUBLIC OF IRAN

## Abstract

**Objective:**

To identify and compare prognostic accuracy of quick Sequential Organ Failure Assessment (qSOFA) score, Systemic Inflammatory Response Syndrome (SIRS) criteria, and National Early Warning Score (NEWS) to predict mortality in patients with suspected sepsis.

**Methods:**

This meta-analysis followed accordance with the recommendations of the Preferred Reporting Items for Systematic Reviews and Meta-Analyses (PRISMA) statement. We searched PubMed, EMBASE, Web of Science, and the Cochrane Library databases from establishment of the database to November 29, 2021. The pooled sensitivity and specificity with 95% CIs were calculated using a bivariate random-effects model (BRM). Hierarchical summary receiver operating characteristic (HSROC) curves were generated to assess the overall prognostic accuracy.

**Results:**

Data of 62338 patients from 26 studies were included in this meta-analysis. qSOFA had the highest specificity and the lowest sensitivity with a specificity of 0.82 (95% CI: 0.76–0.86) and a sensitivity of 0.46 (95% CI: 0.39–0.53). SIRS had the highest sensitivity and the lowest specificity with a sensitivity of 0.82 (95% CI: 0.78–0.85) and a specificity 0.24 (95% CI: 0.19–0.29). NEWS had both an intermediate sensitivity and specificity with a sensitivity of 0.73 (95% CI: 0.63–0.81) and a specificity 0.52 (95% CI: 0.39–0.65). qSOFA showed higher overall prognostic accuracy than SIRS and NEWS by comparing HSROC curves.

**Conclusions:**

Among qSOFA, SIRS and NEWS, qSOFA showed higher overall prognostic accuracy than SIRS and NEWS. However, no scoring system has both high sensitivity and specificity for predicting the accuracy of mortality in patients with suspected sepsis.

## Introduction

In the United States, the sepsis incidence of 5.9% among hospitalized patients, a trend that has been increasing annually [1]. Although the mortality of sepsis has decreased in recent years, it is still the main cause of mortality worldwide [2]. The key strategies for a successful outcome in patients with sepsis are early recognition and timely therapy. However, accurate identification of sepsis is still a problem for clinicians. A reliable method to evaluate sepsis can help clinicians correctly identify sepsis, improve the initial treatment plan of patients, and ultimately improve the survival rate.

Sepsis-1 [3] in 1991 and Sepsis-2 [4] in 2001 suggest that sepsis should be defined as infection with Systemic Inflammatory Response Syndrome (SIRS). The definition of Sepsis-3 [5] was released in 2016 and recommended “Sequential Organ Failure Assessment (SOFA)” or “quick Sequential Organ Failure Assessment (qSOFA)”. The qSOFA score is a simplified score based on the SOFA score, which is said to be more accurate than SOFA in departments outside the intensive care unit (ICU) [6]. In recent years, some studies have tried to evaluate the prognostic accuracy of qSOFA and SIRS. In general, SIRS has high sensitivity but low specificity, and qSOFA has high specificity but low sensitivity in the prognosis of sepsis [7]. In addition, the National Early Warning Score (NEWS) is widely used in the UK as a tool to assess and monitor the clinical status of hospital patients and has the same or higher prognostic accuracy [8]. However, it is unclear which scoring system has higher prognostic accuracy in patients with suspected sepsis.

We included all studies that compared qSOFA, SIRS, and NEWS in suspected sepsis patients and performed a meta-analysis of the available studies to determine the accuracy of these scoring systems in predicting mortality in suspected sepsis patients.

## Method

This meta-analysis was conducted in accordance with the recommendations of the Preferred Reporting Items for Systematic Reviews and Meta-Analyses (PRISMA) statement. The protocol for this review was prospectively registered in INPLASY (number INPLASY202140029)

### Search strategy and selection criteria

We searched PubMed, EMBASE, Web of Science, and the Cochrane Library databases from establishment of the database to November 29, 2021. The search strategy was as follows: (“quick Sequential Organ Failure Assessment” OR “qSOFA”) OR (“Systemic Inflammatory Response Syndrome” OR “SIRS”) OR (“National Early Warning Score” OR “NEWS”) AND (“sepsis”) AND (“mortality”) AND (“emergency department” OR “ED” OR “outside ICU” OR “outside Intensive Care Unit”). Two investigators (Can Wang and Yuerong Zeng) independently screened and included the eligible studies according to the inclusion and exclusion criteria. In case of any disagreement, the study group should discuss and resolve it. The inclusion criteria were the following: (1) the study population was adult patients with suspected or sepsis outside ICU, (2) the purpose was to evaluate or compare the accuracy of qSOFA, SIRS, and NEWS in predicting mortality, (3) a 2 × 2 contingency table (true positives [TP], false positives [FP], false negatives [FN], and true negatives [TN]) can be obtained directly or indirectly through the information in the literature. The exclusion criteria were as follows: review articles, letters, and conference abstracts.

### Data extraction

Two investigators (Can Wang and Yuerong Zeng) independently extracted data from the selected articles. The extracted data were as follows: study characteristics (author, year of publication, country of origin, type of study), patient characteristics (selection criteria of patients, number of patients enrolled, age, sex, setting in which patient was seen), and outcomes (type of measured mortality, cut-off value of qSOFA, SIRS, and NEWS, TP, FP, FN, TN, sensitivity, specificity)

### Study quality assessment

Two investigators (Can Wang and Yuerong Zeng) independently evaluated the quality of included studies according to the Quality Assessment of Diagnostic Accuracy Studies 2 (QUADAS-2) [9]. In case of any disagreement, the study group should discuss and resolve it. The evaluation content mainly consists of four parts: patient selection, index test, reference standard, flow and timing. According to the "yes", "no", or "uncertain" answers to the relevant landmark questions included in each part, the risk of bias can be judged as "low", "high", or "uncertain".

### Statistical analysis

Statistical analysis was conducted using STATA15 and RevMan5.3. The pooled sensitivity and specificity with 95% CIs were calculated using a bivariate random-effects model (BRM) [10]. Hierarchical summary receiver operating characteristic (HSROC) curves were generated to assess the overall prognostic accuracy. Lambda, Theta, and Beta are the estimated parameters of HSROC. Lambda is the natural logarithm of the diagnostic odds ratio (DOR), Theta is the mean of the log of sensitivity and the log of 1-specificity, Beta is the parameter that defines the shape of the summary curve [11]. The Beta value significant difference from zero indicates that the curve is asymmetric, which is not suitable for calculating the pooled value of accuracy [11]. A beta value equal to or close to zero indicates that the curve is symmetrical, and lambda can be used to evaluate the overall prediction accuracy [11]. The post-test probability was assessed by Fagan’s nomogram.

The heterogeneity was evaluated by the *I*^*2*^ test. If *I*^*2*^ ≤ 50%, P ≥ 0.1, the heterogeneity among the studies was acceptable; If *I*^*2*^ > 50%, P < 0.1, the heterogeneity among the studies is significant, and the source of heterogeneity should be analyzed. The threshold effect was not judged because the Stata Midas module was used for statistical analysis. Univariate meta-regression and subgroup analysis were used to explore the source of heterogeneity. Deek’ s funnel plot was made to assess the publication bias.

## Results

### Search results and description of studies

**Fig 1:**
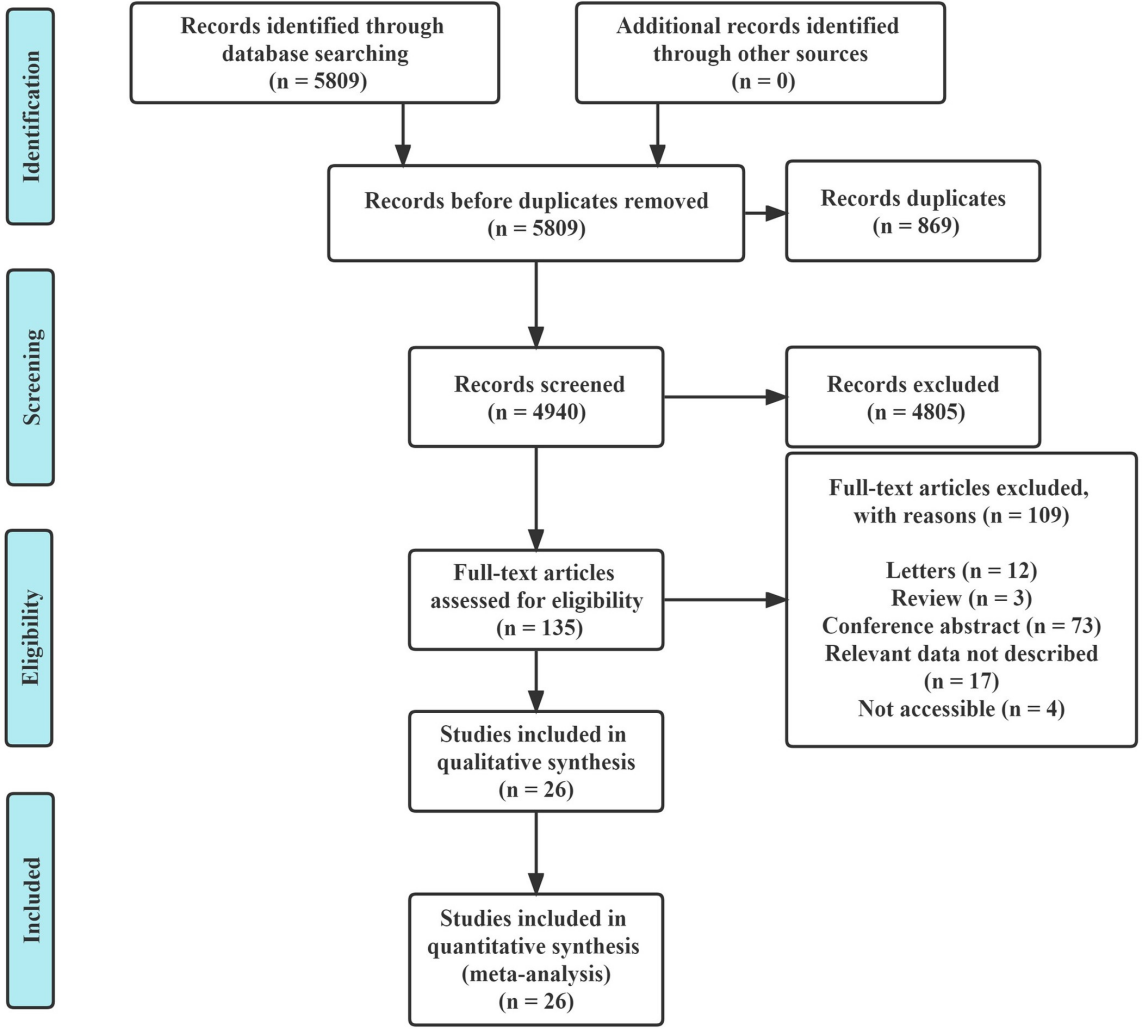
Flow diagram of the study selection process.

5809 articles were found in the initial search, repetitive articles were deleted and 135 articles were reviewed after careful evaluation of abstracts. 109 studies were excluded, of which 12 were letters, 3 were reviews, 73 were conference abstracts, 17 were unable to obtain relevant data, and 4 were unable to accessible. We attempted to contact the manuscript authors to retrieve the 17 articles for which relevant data was not available and 4 articles for which unable to accessible, but there was still no response. 26 studies [8, 12–36] were included according to the inclusion and exclusion criteria (Fig 1). The basic characteristics of the 26 included studies are shown in Table 1. Eight studies [8, 12, 15, 17, 20, 24, 30, 35] were prospective studies and the others [13, 14, 16, 18, 19, 21–23, 25–29, 31–34, 36] were retrospective studies. Five studies [12, 15, 23, 24, 33] were multi-center studies, and the others [8, 13, 14, 16–22, 25–32, 34–36] were described as single-center. The average age varied between 57 and 81, and the proportion of men varied between 46% and 65% in these studies. There were 24 studies [12–17, 18–22, 24–36] including qSOFA, 12 studies [12–15, 17, 19, 20, 25, 26, 30, 32, 36] including SIRS and 11 studies [8, 12–14, 16, 19, 23, 25, 26, 30, 32] including NEWS. The cut-off value of NEWS was 2 in one study [19], 5 in three studies [13, 23, 30], 6 in two studies [12, 25], 7 in two studies [14, 26], and 8 in three studies [8, 16, 32].

**Table 1:** Characteristics of included studies.

Author/Year	Country	Study design	Setting	Patients	Measured mortality	Sample	Male	Age(mean/median)	Cut-off		
						(n)	(%)		qSOFA	SIRS	NEWS
Szakmany/2017 [12]	UK	Prospective	ED or acute in-patient ward	Suspected sepsis	30-day mortality	380	47.4	74	2	2	6
Goulden/2018 [13]	UK	Retrospective	Outside ICU	Suspected sepsis	in-hospital mortality	1818	51.3	58	2	2	5
Brink/2019 [14]	the Netherlands	Retrospective	ED	Suspected sepsis	30-day mortality	8204	55.8	57	2	2	7
Kim/2019 [15]	Korea	Prospective	ED	Severe sepsis or Septic shock	28-day mortality	928	59.5	70.1	2	2	-
Pong/2019 [16]	Singapore	Retrospective	ED	Sepsis	in-hospital mortality	364	49.2	72.8	2	-	8
Sivayoham/2019 [17]	UK	Prospective	ED	Suspected sepsis	in-hospital mortality	1078	50.6	70	2	2	-
Almutary/2020 [8]	Saudi Arabia	Prospective	ED	Suspected sepsis	in-hospital mortality	444	48.2	58.7	-	-	8
Abdullah/2020 [18]	Denmark	Retrospective	ED	Sepsis	in-hospital mortality	434	56.7	70	2	-	-
Boonmee/2020 [19]	Thailand	Retrospective	ED	Sepsis	in-hospital mortality	436	-	-	2	2	5
Caramello/2020 [20]	Italy	Prospective	ED	Sepsis	60-day mortality	469	56.9	73	2	2	-
Guarino/2020 [21]	Italy	Retrospective	ED	Sepsis or septic shock	in-hospital mortality	1001	46.2	79.4	2	-	-
Guirgis/2020 [22]	USA	Retrospective	ED	Sepsis	in-hospital mortality	3297	48.9	59	2	-	-
Hargreaves/2020 [23]	UK	Retrospective	ED	Suspected sepsis	30-day mortality	1233	56.7	79	-	-	5
Mearelli/2020 [24]	Italy	Prospective	ED	Suspected sepsis	30-day mortality	828	51.3	81	2	-	-
Phungoen/2020 [25]	Thailand	Retrospective	ED	Suspected sepsis	in-hospital mortality	8177	52.3	62	2	2	6
Wattanasit/2020 [26]	Thailand	Retrospective	ED	Sepsis	in-hospital mortality	706	54.1	66	2	2	7
Xia/2020 [27]	China	Retrospective	ED	Sepsis	28-day mortality	821	64.3	60	2	-	-
Zhou/2020 [28]	China	Retrospective	ED	Sepsis	28-day mortality	336	63.4	76	-	-	2
Devia Jaramillo/2021 [29]	Colombia	Retrospective	ED	Suspected sepsis	in-hospital mortality	179	50.3	77	2	-	-
Oduncu/2021 [30]	Turkey	Prospective	ED	Suspected sepsis	in-hospital mortality	463	59.2	63	2	2	5
Prasad/2021 [31]	California	Retrospective	ED	Suspected sepsis	in-hospital mortality	23837	53.8	62	2	-	-
Ruangsomboon/2021 [32]	Thailand	Retrospective	ED	Suspected sepsis	in-hospital mortality	1622	48.9	72.6	2	2	8
Shi/2021 [33]	China	Retrospective	ED	Sepsis	in-hospital mortality	574	65.2	71.3	2	-	-
Sivayoham/2021 [34]	UK	Retrospective	ED	Suspected sepsis	in-hospital mortality	2594	53.2	73	2	-	-
Suttapanit/2021 [35]	Thailand	Prospective	ED	Suspected sepsis	28-day mortality	1139	46.4	70	2	-	-
Kilinc Toker/2021 [36]	Turkey	Retrospective	ED	Sepsis	in-hospital mortality	976	47.3	72.5	2	2	-

*qSOFA = quick Sequential Organ Failure Assessment; SIRS = Systemic Inflammatory Response Syndrome; NEWS = National Early Warning Score.

### Quality assessment of studies

**Fig 2:**
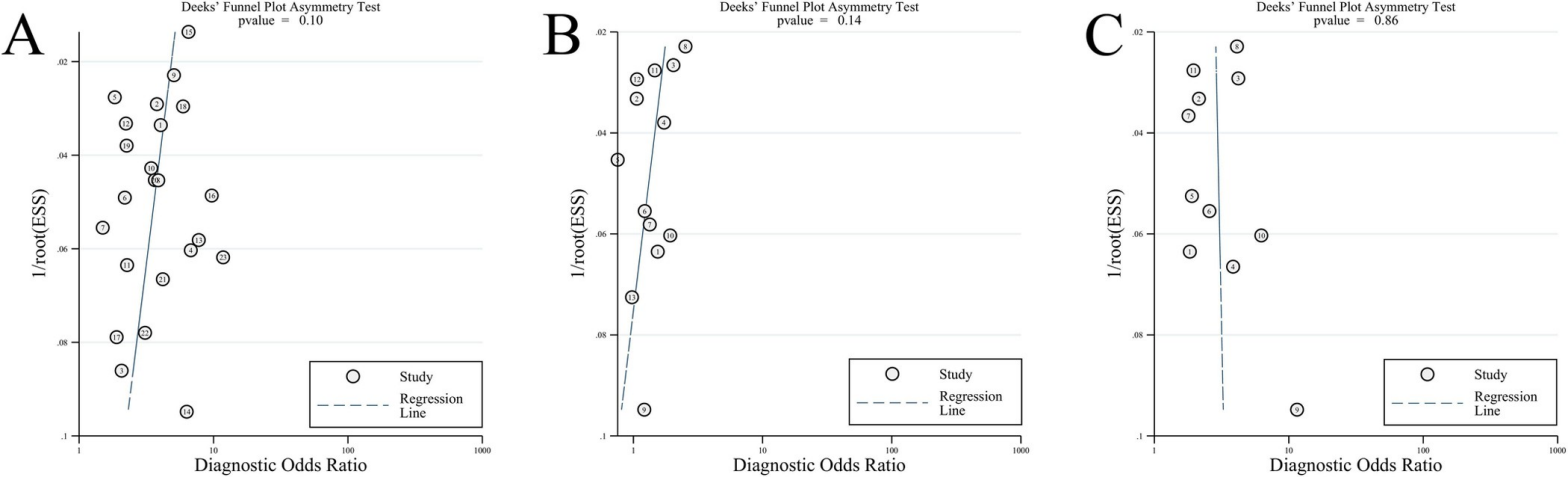
Deek’ s funnel plot for publication bias. A: qSOFA; B: SIRS; C: NEWS.

The individual and overall quality assessment results of the 26 included studies were shown in S1 Fig. Overall, the included studies showed that there were risks in two of the four areas. Except for the study of Brink, et al [14], all other studies included consecutive or random cases, avoiding inappropriate exclusion. Because the cut-off values of the 11 studies [8, 16, 17, 25, 26, 28, 32–36] were not determined in advance, there were high risks in the index test. Dick’s funnel plot suggested that there was no publication bias (Fig 2).

### Data synthesis and meta-analysis

#### Predictive validity of qSOFA

**Fig 3:**
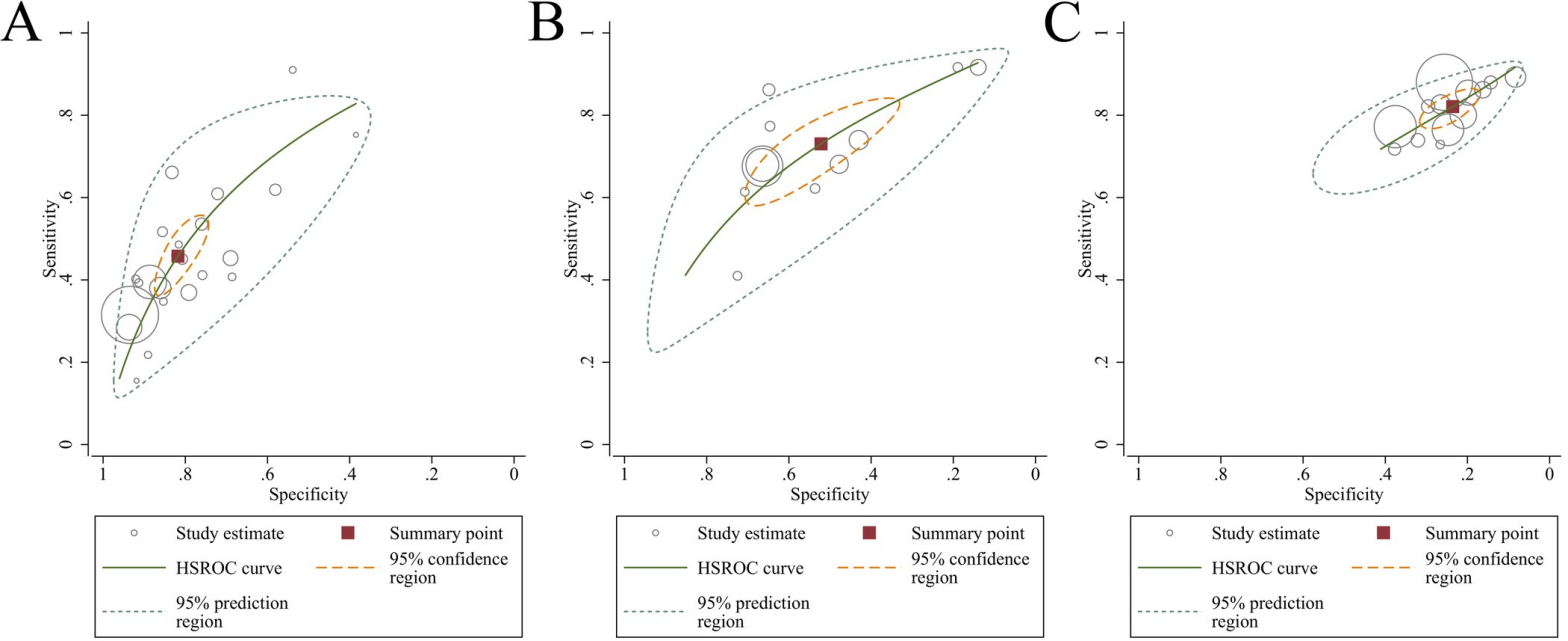
Hierarchical Summary Receiver Operating Characteristic (HSROC) curve for predicting mortality in patients with suspected sepsis. A: qSOFA; B: SIRS; C: NEWS.

**Fig 4:**
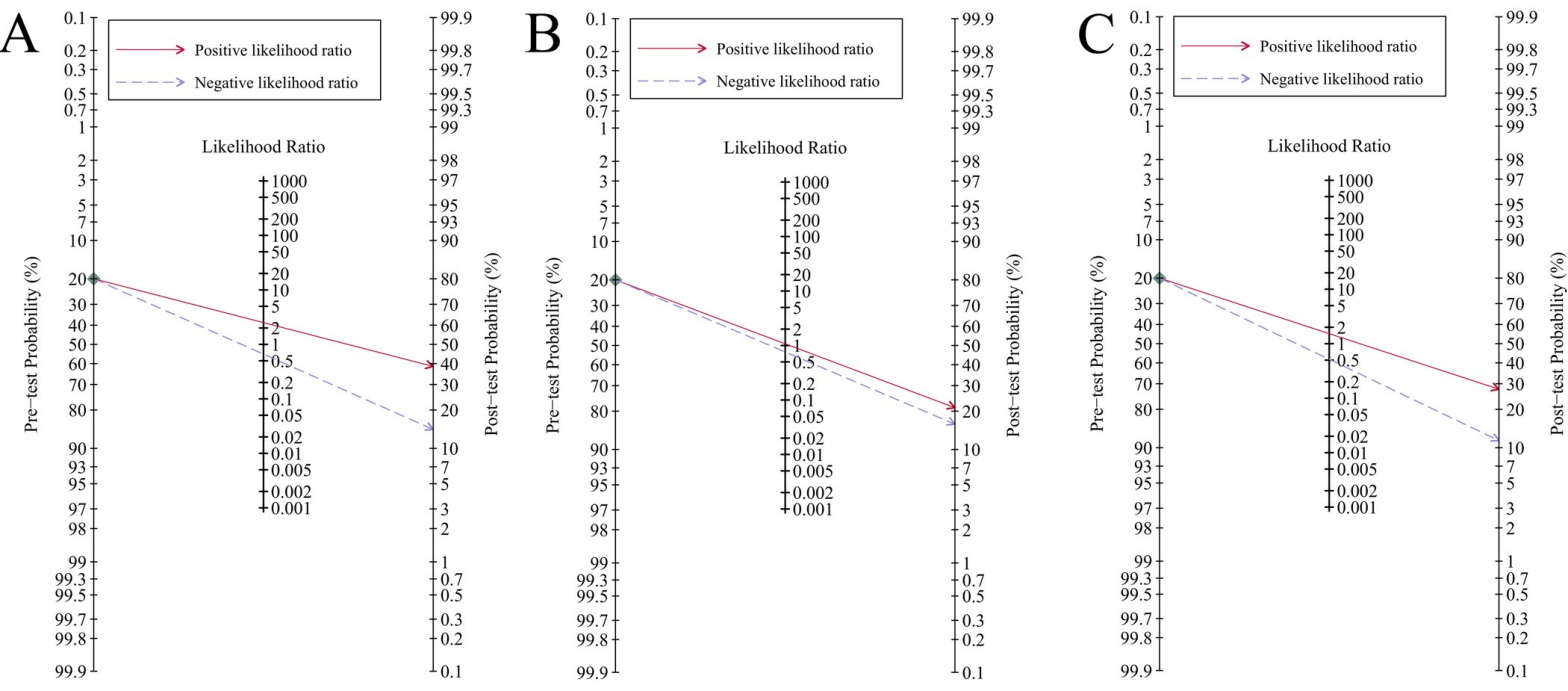
Fagan’ s nomogram for pre-test and post-test probability of mortality in patients with suspected sepsis. A: qSOFA; B: SIRS; C: NEWS.

Data of 60661 patients in 24 studies were associated with the accuracy of qSOFA in predicting mortality. The pooled sensitivity and specificity were 0.46 (95% CI: 0.39–0.53) and 0.82 (95% CI: 0.76–0.86), respectively (S2 Fig). The *I*^*2*^ of sensitivity and specificity were 95.10% and 99.25% indicating significant heterogeneity among the studies. The heterogeneity sources were analyzed by meta-regression and subgroup analyses, and the results were shown in S5A Fig. The types of studies (*P*<0.05), the types of patients (*P*<0.05), and the types of mortality measured (*P*<0.01) may be the source of heterogeneity. The HSROC curve and the estimated parameters of qSOFA were shown in Fig 3A and Table 2. Beta was 0.12 (Z = -0.74, *P* = 0.459), which indicates that the curve is symmetric. Lambda was 1.23 (95% CI: 0.91–1.55), which represents corresponding the DOR of 3.79. Fagan’ s nomogram (Fig 4A) showed that qSOFA increased the possibility of mortality in patients with suspected sepsis to 39% when the pretest probability of mortality was 20%.

**Table 2:** The estimated parameters of HSROC.

	Lambda (95%CI)	Theta (95%CI)	Beta		
			Beta (95%CI)	*Z*	*P*
qSOFA	1.23(0.91–1.55)	-0.80(-1.10–0.49)	0.12(-0.18–0.43)	0.79	0.427
SIRS	0.79(0.09–1.50)	1.40(1.14–1.65)	0.32(-0.17–0.82)	1.28	0.200
NEWS	1.18(0.87–1.50)	0.51(0.03–1.00)	0.20(-0.155–0.56)	1.11	0.267

#### Predictive validity of SIRS

The pooled sensitivity and specificity of SIRS for predicting mortality in suspected sepsis patients were 0.82 (95% CI: 0.78–0.85) and 0.24 (95% CI: 0.19–0.29), respectively (S3 Fig). The HSROC curve and the estimated parameters of SIRS were shown in Fig 3B and Table 2. Significant heterogeneity between studies can be observed in the combined results of sensitivity and specificity (*I*^*2*^ = 77.08% and *I*^*2*^ = 98.32%). Meta-regression analysis and subgroup analysis showed that the heterogeneity of sensitivity was caused by the types of studies, patients’ settings, the types of patients, and the types of mortality measured, and the heterogeneity of specificity was caused by the types of study and types of patients (S5B Fig). Beta was 0.32 (Z = 1.28, *P* = 0.200) and Lambda was 0.79 (95% CI: 0.09–1.50). Fagan’s nomogram (Fig 4B) showed that SIRS increased the possibility of mortality in patients with suspected sepsis to 21% when the pretest probability of mortality was 20%.

#### Predictive validity of NEWS

The pooled sensitivity and specificity of NEWS for predicting mortality in suspected sepsis patients were 0.73 (95%CI, 0.63–0.81) and 0.52 (95%CI, 0.39–0.65), respectively (S4 Fig). Heterogeneity among studies was considered substantial in the analyses of sensitivity and specificity, with *I*^*2*^ values of 92.50% for sensitivity and 99.47% for specificity. Meta-regression analysis and subgroup analysis showed that the heterogeneity of sensitivity among the studies came from the types of studies (S5C Fig). Beta was 0.20 (Z = 1.11, *P* = 0.267), which indicates that the curve is symmetric. Lambda was 1.18 (95% CI: 0.87–1.50), which represents corresponding the DOR of 2.96 (Fig 3C and Table 2). Fagan’s nomogram (Fig 4C) showed that NEWS increased the possibility of mortality in patients with suspected sepsis to 28% when the pretest probability of mortality was 20%.

#### Performance comparison of qSOFA, SIRS and NEWS

**Fig 5:**
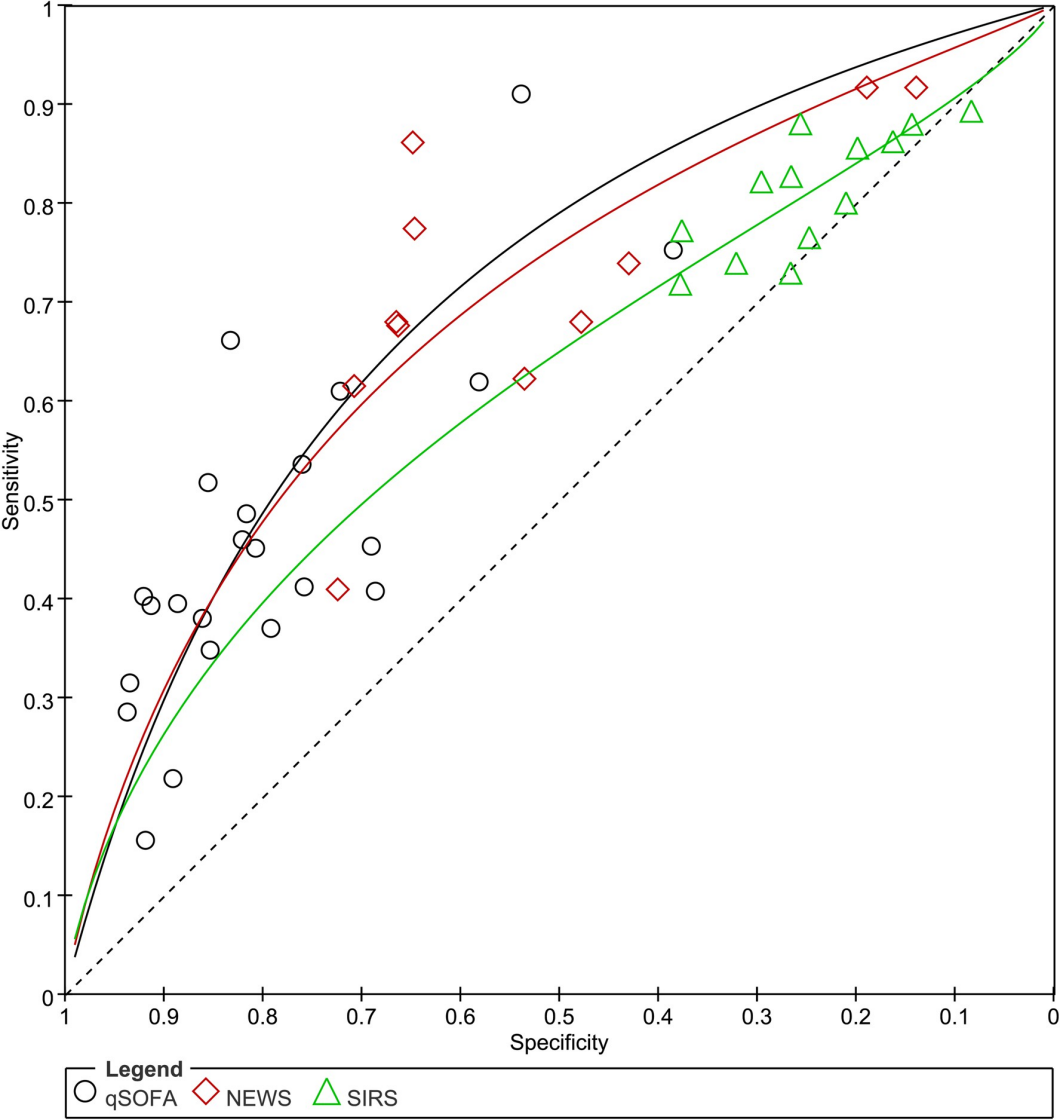
Direct comparison among qSOFA, SIRS, and NEWS in predicting the accuracy of mortality in patients with suspected sepsis.

The performance of the qSOFA, SIRS and NEWS in predicting mortality in patients with suspected sepsis were presented in Fig 5 and Table 3. In direct comparisons, qSOFA showed higher overall prediction accuracy than SIRS and NEWS with the diagnostic odds ratio (DOR). In addition, the AUC in qSOFA and NEWS were higher than in SIRS. qSOFA provided the highest specificity for predicting mortality followed by NEWS and SIRS. On the contrary, the SIRS provided the highest sensitivity for predicting mortality followed by NEWS and qSOFA. qSOFA showed better post-test probability than SIRS and NEWS, representing patients with suspected sepsis met qSOFA the greater chance of die.

**Table 3:** Pooled performance characteristics of qSOFA, SIRS, and NEWS for predicting mortality in patients with suspected sepsis.

	Sensitivity (95%CI)	Specificity (95%CI)	AUC (95%CI)	DOR	Post-test probability
qSOFA	0.46(0.39–0.53)	0.82(0.76–0.86)	0.69(0.65–0.73)	3.79	39%
SIRS	0.82(0.78–0.85)	0.24(0.19–0.29)	0.63(0.58–0.67)	1.42	21%
NEWS	0.73(0.63–0.81)	0.52(0.39–0.65)	0.69(0.65–0.73)	2.96	28%

## Discussion

This is the first meta-analysis comparing the prognostic accuracy of qSOFA, SIRS, and NEWS to predict mortality in patients with suspected sepsis. Our meta-analysis identified 26 clinical studies, including 62338 patients with suspected sepsis. However, no scoring system has both high sensitivity and specificity for predicting the accuracy of mortality in patients with suspected sepsis. These scoring systems have their advantages and disadvantages.

qSOFA is a bedside assessment tool recommended by the Third International Sepsis Consensus Definitions Task Force used to assess patients with suspected sepsis outside the ICU [5]. Our findings suggest that qSOFA shows the highest overall prediction accuracy of mortality and has high specificity. Therefore, qSOFA is of great value in predicting the mortality of patients with suspected sepsis. It can more accurately identify patients with a high risk of death than the other two scoring systems. However, our results show that qSOFA has low sensitivity, which means that false negative is high and it is easy to miss or delay treatment. The reasons for the low sensitivity of qSOFA may be as follows. On the one hand, the supporting paper [6] derived and tested among critically ill patients and there will be deviation and sensitivity will be decreased for the general patient population. On the other hand, qSOFA ≥ 2 is too strictly and patients will be in a late disease state with a worse prognosis using it [37]. Due to the lethality of sepsis, a screening mechanism showing high sensitivity is needed [38]. Some studies have proposed that reducing the qSOFA cut-off to 1 or combined with lactate levels can improve the sensitivity [39].

It is universally acknowledged that any scoring system used to determine sepsis should tend to be higher sensitivity rather than specificity because the cost of delaying or missing treatment caused by false negatives is much greater than the cost of unnecessary antibiotics caused by false positives [13]. We found that SIRS had the highest sensitivity among the three scoring systems. However, SIRS is too poor to predict mortality in patients with suspected sepsis. Whether direct comparison or indirect comparison, SIRS has the lowest prediction ability. SIRS is more suitable as a screening tool for early care and the prevention of missed diagnoses.

NEWS is widely recommended to identify patients at risk of deterioration, which was launched by the Royal College of Physicians (RCP) in 2012 [40]. NEWS has the strongest ability to identify patients at risk of deterioration compared with other Early Warning Score (EWS) [41]. Meanwhile, more and more studies have proved that NEWS is a promising scoring system and can be used as an alternative screening tool for patients with suspected sepsis. In this meta-analysis of patients with suspected sepsis, we found that NEWS is slightly worse than qSOFA in terms of overall prediction ability, but avoids the extremely low sensitivity of qSOFA and has both an intermediate sensitivity and specificity. The strength of the NEWS is that it can be calculated based on physiological parameters alone, which is easier to implement than the other two scoring systems [42].

Our research also has some limitations. On the one hand, there is significant heterogeneity in our meta-analysis. The included studies were different types of studies (prospective or retrospective), different outcome indicators (in-hospital mortality or 30 / 28 / 60-day mortality), and different types of patients (sepsis or suspected sepsis). The above points are the sources of heterogeneity. On the other hand, the cut-off values of the included studies are different, and the cut-off values of some studies are not determined in advance. A predefined cut-off value help to reduce the sensitivity and specificity bias that may result from this data-driven method [43].

## Conclusion

In conclusion, our results indicate that qSOFA showed higher overall prediction accuracy of mortality than SIRS and NEWS. The three scoring systems have limitations as a tool for predicting mortality in patients with suspected sepsis. A scoring system with both high sensitivity and specificity needs to be studied in the future.

## Supporting information

S1 Fig Summary of methodological quality in the included studies. (JPG)

S2 Fig Forest plot for sensitivity and specificity of qSOFA for predicting mortality in suspected sepsis patients. (JPG)

S3 Fig Forest plot for sensitivity and specificity of SIRS for predicting mortality in suspected sepsis patients. (JPG)

S4 Fig Forest plot for sensitivity and specificity of NEWS for predicting mortality in suspected sepsis patients. (JPG)

S5 Fig Univariate meta-regression and subgroup analysis for sensitivity and specificity. Factors with asterisk are potential sources of heterogeneity. A: qSOFA; B: SIRS; C: NEWS.
(JPG)

S1 File PRISMA 2009 checklist. (DOC)
