# Supplementary figures and images for: A comparison of qSOFA, SIRS and NEWS in predicting the accuracy of mortality in patients with suspected sepsis: A meta-analysis
Source: PLoS One. 2022 Apr 15;17(4):e0266755. doi: 10.1371/journal.pone.0266755 (PMC9012380; doi:10.1371/journal.pone.0266755)

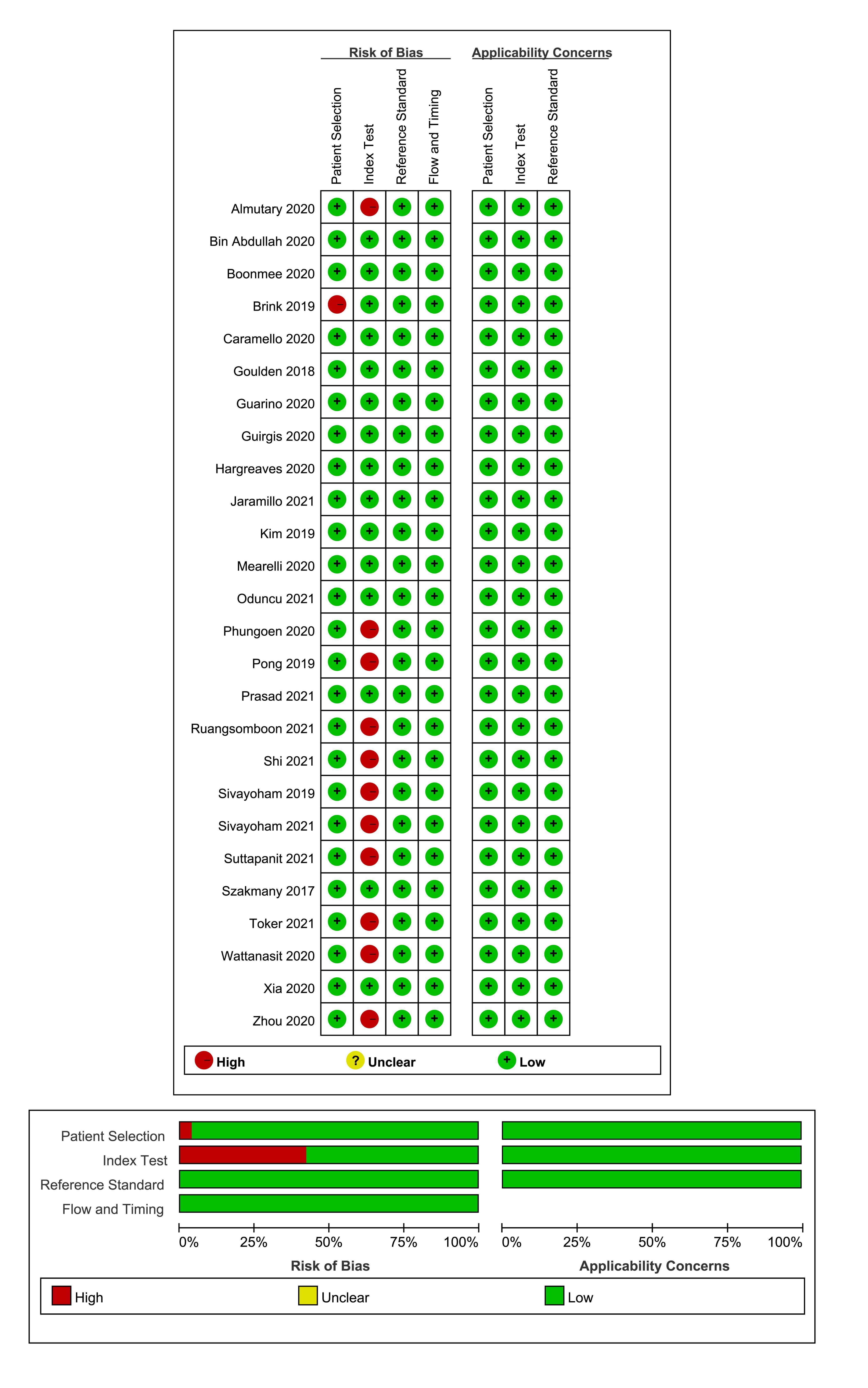

Supplement: S1 Fig — (JPG) [file pone.0266755.s001.jpg]

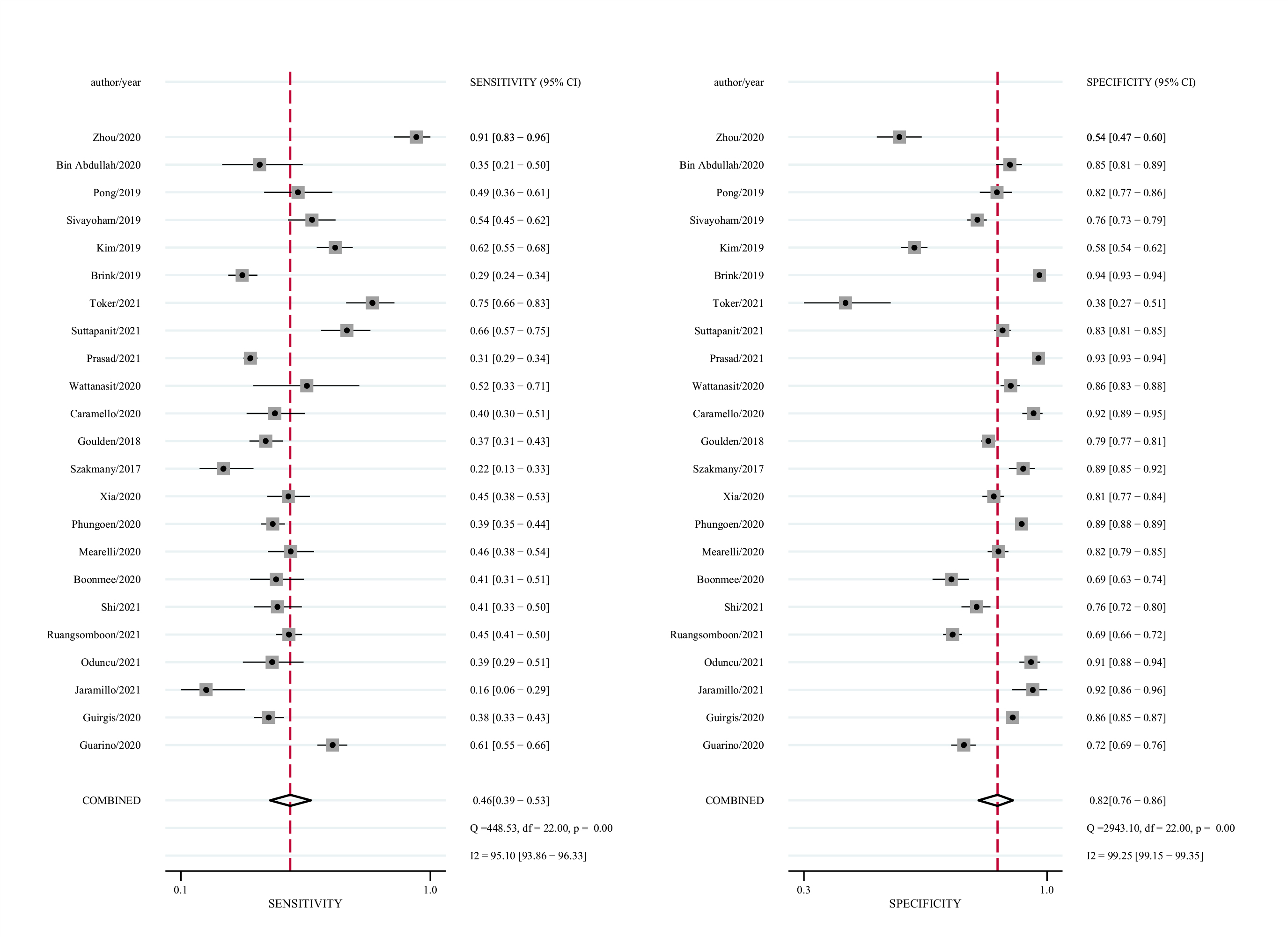

Supplement: S2 Fig — (JPG) [file pone.0266755.s002.jpg]

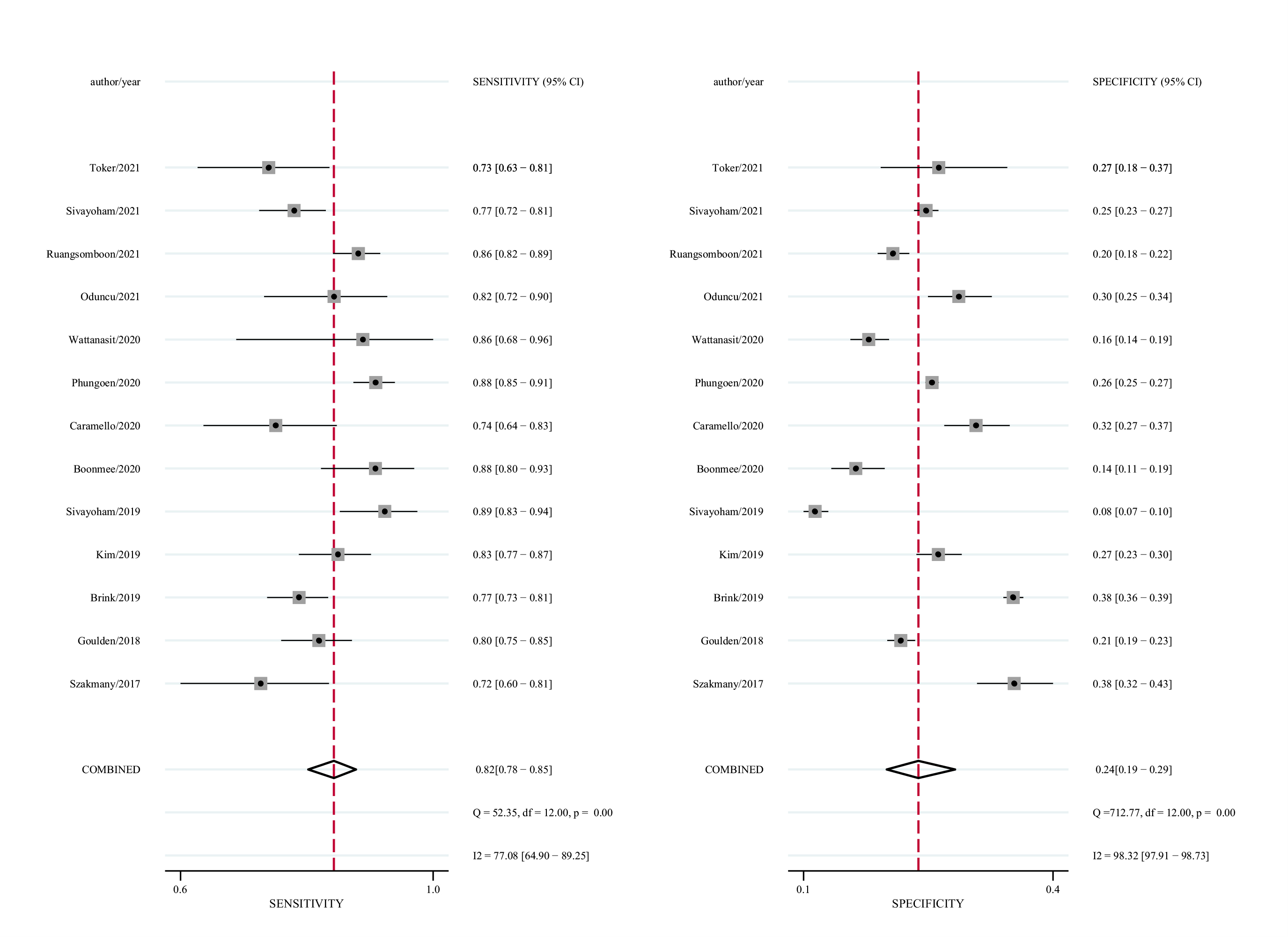

Supplement: S3 Fig — (JPG) [file pone.0266755.s003.jpg]

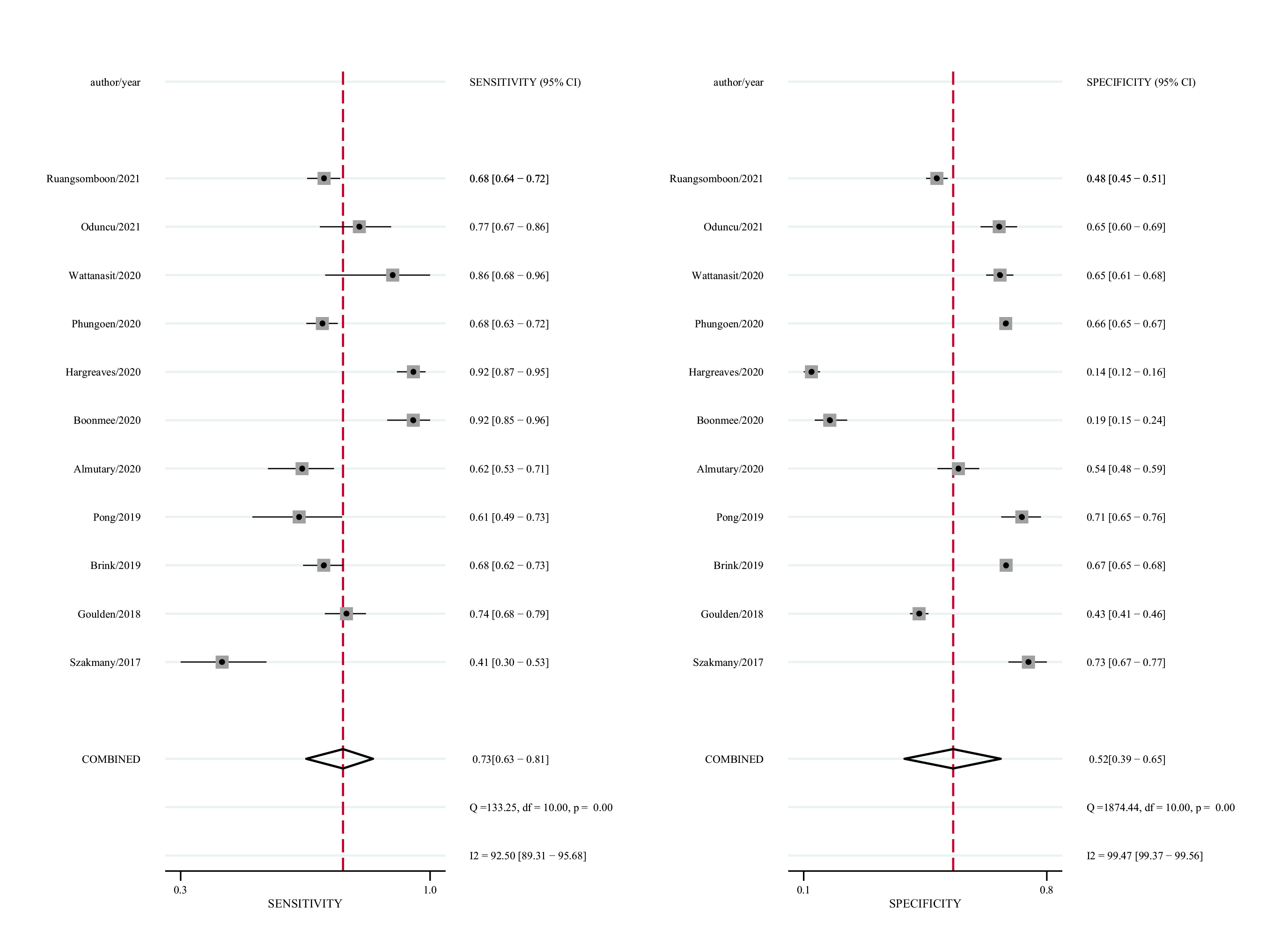

Supplement: S4 Fig — (JPG) [file pone.0266755.s004.jpg]

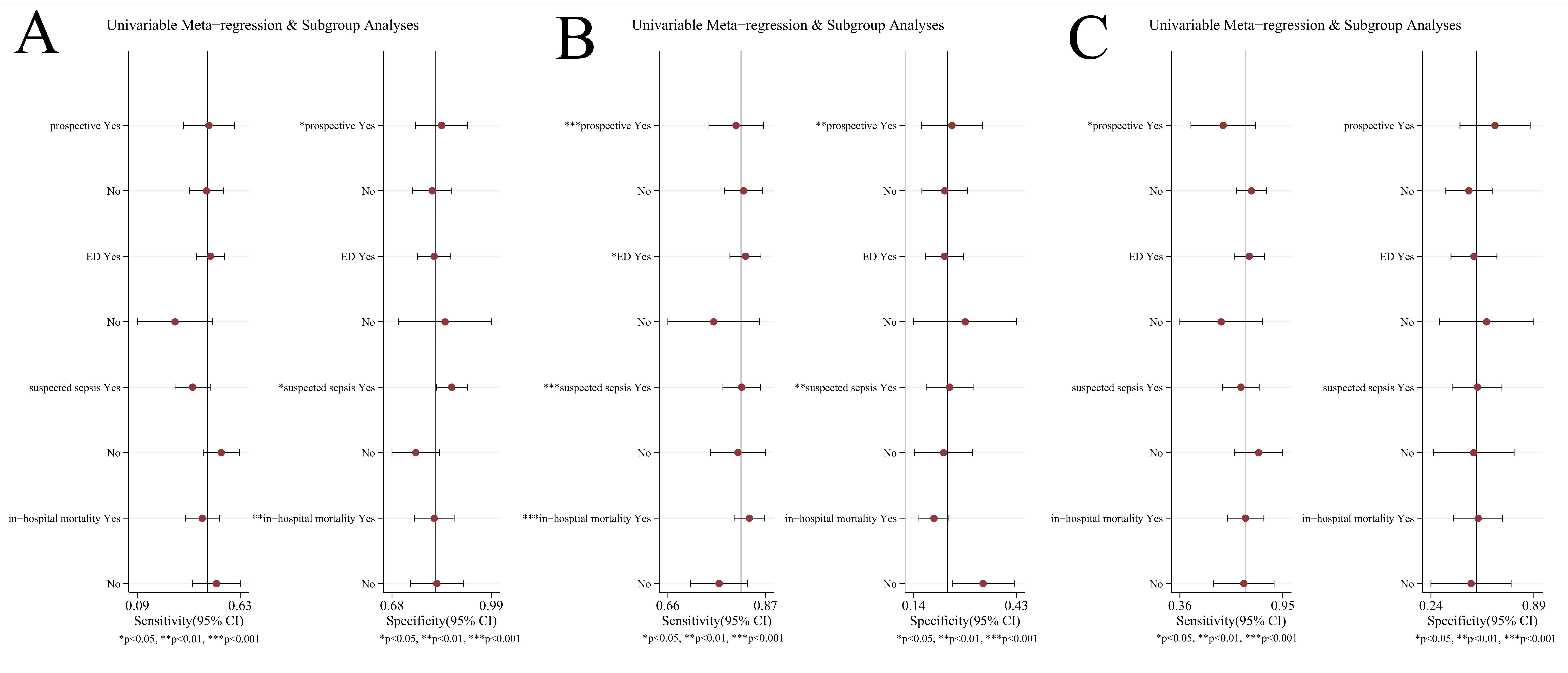

Supplement: S5 Fig — Factors with asterisk are potential sources of heterogeneity. A: qSOFA; B: SIRS; C: NEWS. (JPG) [file pone.0266755.s005.jpg]
